# Numerical Simulation of Deformation in Hot Runner Manifold

**DOI:** 10.3390/mi14071337

**Published:** 2023-06-29

**Authors:** Jae Sung Jung, Sun Kyoung Kim

**Affiliations:** Department of Mechanical System Design Engineering, Seoul National University of Science and Technology, Seoul 01811, Republic of Korea; sungs@seoultech.ac.kr

**Keywords:** injection molding, hot runner, mold, deformation

## Abstract

This study simulated the deformation of a hot runner manifold and nozzle assembly during operation, aiming to address potential leaks and premature failure. Both thermal and mechanical models were used simultaneously to accurately capture system behavior. A simplified set of boundary conditions was proposed for efficient problem-solving. Analysis of the simulation results revealed that thermal deformation posed a risk of catastrophic failures and leaks. Deformation from melt pressure was relatively small compared to thermal loading, not exceeding 12%. The study provided design recommendations based on the simulation findings, guiding the development of hot runner systems for improved reliability.

## 1. Introduction

The demand for mass-produced plastic parts through injection molding is steadily rising. There is also a growing emphasis on achieving high dimensional stability and appearance quality in these products. However, there is increasing pressure to reduce unit prices, making it challenging to maintain profitability in injection production.

Consequently, the injection molding industry is increasingly adopting hot runner systems to enhance process efficiency and cost-effectiveness. The tight nature of the molding process has led to growing concerns regarding material waste. Furthermore, modern injection molded parts strive to minimize the need for reprocessing scraps.

At times, the construction practices of hot runner systems deviate from established theories or protocols due to the differing intuitions of tooling designers or manufacturers [1,2,3]. Mold-making companies traditionally have their own approaches to managing dimensions, clearances, and roughness. While these practices may vary, they should still adhere to scientific principles.

The difficulties with hot runners arise from the fact that they are machined and assembled at room temperature but must be operated at high temperatures. Moreover, this assembly includes a connection by bolts, and this connection by bolts constrains deformation at high temperatures to an unexpected form. If bolts are applied only for strong fastening without a close examination based on mechanical principles, thermal deformation occurs mainly in areas where fastening is weak. Due to the nature of the hot runner, a huge temperature gradient is generated between the parts, which causes strain of a size that cannot be handled only by simple fastening. In a hot runner, the plates are kept cold, but the manifolds and nozzles are exposed to difficult thermal conditions that must be heated close to the melt temperature. Therefore, the fastening between the high-temperature part and the low-temperature part should be performed in consideration of thermal expansion in an operating situation. At this time, where and how to fasten is an important task, and for this, it is essential to three-dimensionally predict the deformation. 

This work presents a novel method for simulating the structural deformation of hot runner systems. While previous studies have explored deformation in injection molds [4,5,6,7], there is limited research on the deformation of hot runner systems. Notably, only one study found has addressed nozzle deformation [8]. Existing studies on hot runners have primarily focused on efficient melt delivery [9,10] and sequential gating [11,12]. Some investigations have also examined pressure drop, velocity, and heat transfer in the runner flow of melt [13,14,15]. 

This study aims to develop a suitable thermal and mechanical model for the hot runner system, considering its structural aspects. The model should achieve a balance between accuracy and simplicity. To accurately capture the behavior of the hot runner system, a three-dimensional model is crucial. The model primarily focuses on the manifold and nozzle, which are essential components of the system. Detailed modeling is carried out for these parts, while the remaining components are represented in a more general manner.

While the simulation does not incorporate the melt flow due to its minimal impact on deformation, it assumes a static pressure applied to the contact surface with the melt. The simulation results obtained under these conditions will be presented and analyzed to provide valuable insights for manifold design.

## 2. Simulation 

### 2.1. Hot Runner System

**Figure 1:**
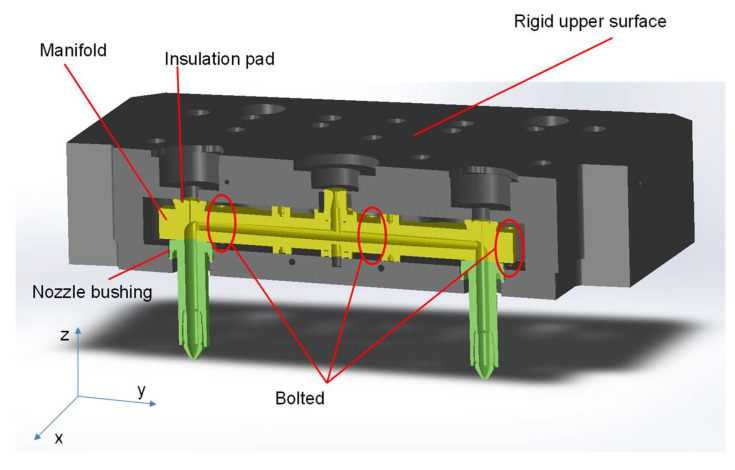
Overall structure of the hot runner system.

This study focuses on a simplified hot runner system consisting of a manifold connected to two nozzles, as illustrated in Figure 1. For simplicity, the upper plate, which is subjected to the moving plate, is assumed to be rigid. While the deformation of the upper plate can be a concern, this work does not specifically address it. The manifold is identified as the most problematic component in the conventional hot runner setup.

Designing the manifold to achieve both thermal insulation and structural integrity under high melt pressure poses a significant challenge in hot runner systems. Fixing the manifold to the mold plates without compromising thermal performance is particularly challenging. Over the years, mold manufacturers and individuals have patented various ideas related to this issue [16,17,18,19,20,21], with a particular focus on sealing [20] and spring force [21]. However, these approaches are not widely adopted due to cost, design restrictions, and reliability concerns. Consequently, reducing deformation through proper design becomes imperative in addressing this challenge.

The design depicted in Figure 1 represents a typical shape of a hot runner system, as documented in [1,2,3]. The dimensions used in this work were determined by the authors, following a design guide [2]. As illustrated, the design prioritizes minimizing contact with surrounding structures. While not explicitly shown, the structure incorporates three or more bolts for enhanced structural stability. However, due to its inherent weakness compared to the surrounding members, the design is susceptible to deformation, and the number of fastenings is limited to mitigate this vulnerability.

The number of fastenings in the manifold can contribute to the formation of temperature gradients, leading to thermal bridges. These bridges can disrupt temperature control and create uneven heating, resulting in hot and cold spots. Moreover, the constraints imposed by boltings and bushings can induce deformation in the overall structure. One of the critical consequences of manifold deformation is the failure of heat control, as it can cause melt leakage due to misalignment of the nozzle and manifold connections.

Hence, it is crucial to ensure intimate contact between the nozzle and manifold interface. It is important to effectively control the deformation of the manifold to ensure that the interface does not become excessively deformed. The bending deformation occurs due to the constraints, causing the manifold to deviate from parallel alignment with the surrounding plates. Unfortunately, in many cases, attempts to address resin leaks involve mistakenly tightening the parts further. In this study, we will investigate and discuss the characteristics of the associated deformation, using simulations as a basis.

### 2.2. Simple Deformation Model

**Figure 2:**
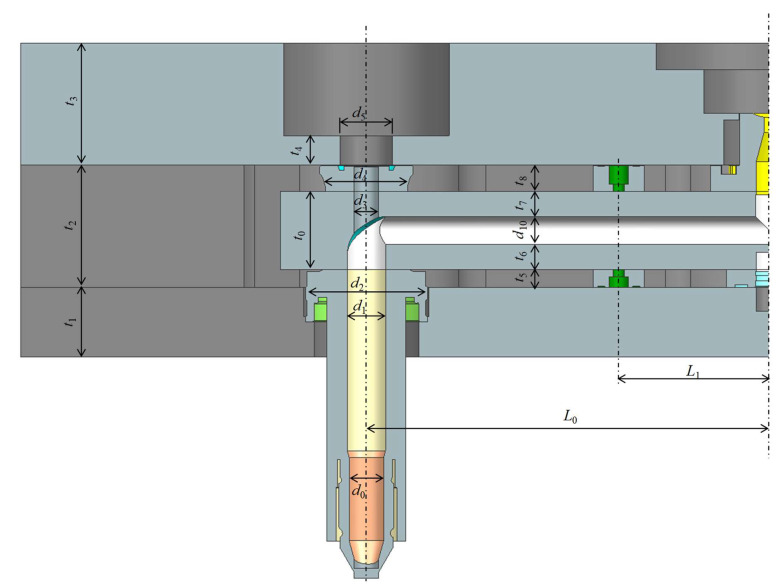
Major geometric parameters of the hot runner at the operational temperature.

Figure 2 shows the dimensional parameters of the considered hot runner system. Refer to the figure for *t*_i_, *s*_i_, *L*_i_, and *d*_i_. Some of the detailed dimensions around the nozzle and the sprue are suppressed. The dimension in the figure is for the operational condition. Let us denote the dimensions at the reference temperature of machining by $t_{i}^{o}$, $s_{i}^{o}$, $L_{i}^{o}$ and $d_{i}^{o}$, respectively. As mentioned earlier, the dimensions change significantly while heating up. To minimize the corresponding deformation, intentional gaps are required. The vertical gap is defined by

(1) $$t_{gap}^{o}=t_{2}^{o}-\left(t_{0}^{o}+t_{5}^{o}+t_{8}^{o}\right)$$

At the operation temperature, the gap would change to

(2) $$t_{gap}=t_{2}-\left(t_{0}+t_{5}+t_{8}\right)$$

Ideally, $t_{gap}$ should be set to zero. Considering the thermal expansion, $t_{gap}^{o}\geq t_{gap}$ should be met. The difference can be approximated by

(3) $$t_{gap}^{o}-t_{gap}=\alpha_{2}\Delta T_{2}t_{2}^{o}-\left(\alpha_{0}\Delta T_{0}t_{0}^{o}+\alpha_{5}\Delta T_{5}t_{5}^{o}+\alpha_{8}\Delta T_{8}t_{8}^{o}\right)$$

where $\alpha_{i}$ and $\Delta T_{i}$ represents the CTE (coefficient of thermal expansion) and the temperature difference at the *i*-th section.

### 2.3. Mechanical Model

This work assumes a simple steady simulation of an elastic deformation involving linear thermal expansion. The Equation of equilibrium is

(4) $$\frac{\partial \sigma_{ij}}{\partial x_{j}}+f_{i}=0$$

where $\sigma_{ij}$ is the stress tensor and $f_{i}$ is the body force in the $x_{i}$ direction. Note that the position variable $x_{i}$ constitutes a vector **x**. Then, Hooke’s law combined with thermal strain describes the stress as

(5) $$\sigma_{ij}=C_{ijkl}(\varepsilon_{kl}-\varepsilon_{kl}^{T})$$

where $C_{ijkl}$ or C is the fourth-order tensor of material stiffness. The stiffness tensor in three-dimension can be described as

(6) $$C={\left[\begin{array}{cccccc} 1/E & -v/E & -v/E & 0 & 0 & 0\\ -v/E & 1/E & -v/E & 0 & 0 & 0\\ -v/E & -v/E & 1/E & 0 & 0 & 0\\ 0 & 0 & 0 & 1/G & 0 & 0\\ 0 & 0 & 0 & 0 & 1/G & 0\\ 0 & 0 & 0 & 0 & 0 & 1/G \end{array}\right]}^{-1}$$

where *E*, *G*, and *ν* are Young’s modulus, shear modulus, and Poisson’s ratio, respectively. The thermal strain is of the form

(7) $$\varepsilon_{kl}^{T}=\delta_{kl}\alpha \Delta T$$

where α is the CTE in direction k. Here, the temperature difference, ΔT, is defined as

(8) $$\Delta T=T-T_{r}$$

where *T* is the processing temperature at which the hot runner is under operation. $T_{r}$ is the reference temperature where the hot runner structure is not heated. Therefore, the magnitude of the strain that occurs eventually is ultimately determined by this temperature difference.

### 2.4. Thermal Model

In order to determine the temperature field, *T*, the thermal problem by the energy equation should be solved together with a set of proper boundary conditions. The heat transfer in the hot runner system can be simplified to a heat conduction problem of the form

(9) $$\rho c\frac{\partial T}{\partial T}=\nabla k\cdot \nabla T+\dot{q}$$

where the thermal conductivity, *k*, the specific heat capacity, *c*, and the density *ρ* should be specified. In addition, heat generation $\dot{q}$ is introduced to account for electric energy input by heaters during hot runner control. Depending on the manufacturing state of the heater, this heat generation may be non-uniform, but in this study, uniform heat generation was assumed inside the heater.

This is a very stable calculation that can be easily achieved without great numerical concern. However, the imposition of the proper boundary condition is not always easy. The boundaries of exposed air are treated as

(10) $$-k\frac{\partial T}{\partial n}=h\left(T-T_{\infty }\right)+\varepsilon \sigma \left(T^{4}-T_{\infty }^{4}\right)$$

where $T_{\infty }$ is the air temperature and *n* is the direction normal toward the outside of the surface. Moreover, in the domain enclosed by the manifold and the surrounding structures containing it, the surface-to-surface radiation, as well as the convective boundary condition, has been taken into consideration for accurate simulation. The mathematical description of the radiation has been suppressed in this work since it is quite bulky to fully describe here. 

**Figure 3:**
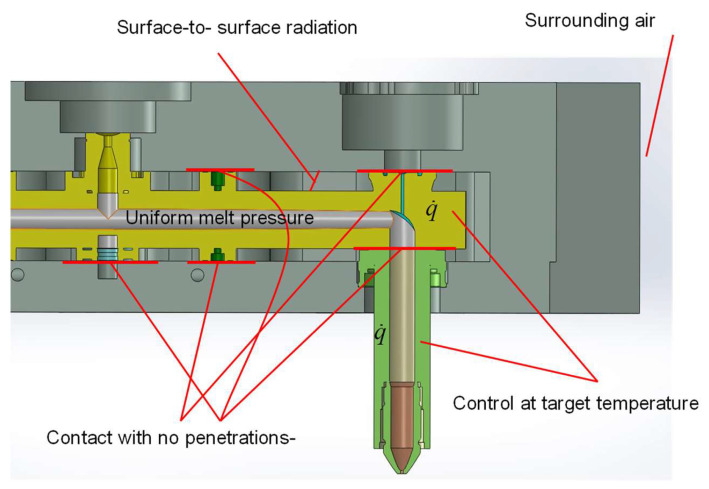
Setup for the simulation.

Figure 3 illustrates the setups used for the simulation described in this work. It should be noted that the presence of polymer melt in the channels is neglected, and the boundaries are assumed to be thermally insulated. Furthermore, each part is considered to be in mechanical contact. This is achieved by applying a contact boundary condition, enabling the simulation of separation between the nozzle and the manifold. To simplify the problem, the temperature of the air between the manifold and the template is approximated as the average value between the manifold control temperature and the set temperature of the template. This method is commonly used to simplify the heat transfer problem, and the resulting temperature is referred to as the film temperature.

### 2.5. Numerical Simulation

This work addresses a conjugate problem by considering both heat conduction and static deformation in the simulation. The heat conduction analysis incorporates boundary conditions that account for both convection heat transfer and surface-to-surface radiation.

Realistically simulating the hot runner system depicted in Figure 1 requires careful attention to its geometric complexity. To achieve an accurate representation, each part constituting the system is modeled individually and assembled using non-penetrating contacts. Assuming the parts are welded would not significantly affect the thermal problem, but it would have a significant impact on the structural analysis. Therefore, non-penetration contact is enforced between the surfaces using a penalty method. The no-penetration contact condition is expressed mathematically as

(11) $$D\left(S_{1},S_{2}\right)\geq 0$$

Here, *D* is the signed distance between the surfaces considering the outward normal vector.

It is important to note that the sprue is treated as blocked and subject to constant static pressure. As mentioned before, no flow is considered in this simulation. Therefore, the sprue is treated under the condition that normal stress is applied to the wall surface. 

This work employs SolidWorks Simulation to accommodate all of the factors described above. SolidWorks Simulation is a viable option for this simulation because it is capable of handling these factors without great burden. The heat generation is estimated by obtaining the integrated energy input required to maintain the target temperature in the simulation system. This has been performed by trial and error.

## 3. Results

### 3.1. Simulation Conditions

The simulation requires several material properties, such as Young’s modulus, Poisson’s ratio, thermal conductivity, and heat capacity. A hot runner assembly is considered built of the AISI H13 tool steel. The manifold and nozzles are maintained at a temperature of *T_p_*, while the plates and space block are at *T_r_*. Moreover, the properties of the insulation pad are obtained from the manufacturer. The values for the simulations are presented in Table 1.

A large injection molding machine such as Engel ES4550, which is capable of 10,000 kN clamping, can generate a melt pressure of up to 218 MPa. The maximum pressure can instantaneously rise over the pressure in the specification. The ratio of possible excess rise is considered to reach 0.382, which is the first number in the set of Fibonacci-derived ratios [22]. In the case of such an accidental 38% rise, the pressure can be as large as 300 MPa. In consideration of this, the entire HRS walls are set subject to 300 MPa.

**Table 1:** Material constants in this simulation [23].

Properties	Plates Space Blocks	Manifold Nozzle	Insulation Pad
*E*	190 GPa	163 GPa	170 GPa
*ν*	0.29	0.32	0.26
α	10.6 µm/m/K	11.8 µm/m/K	12 µm/m/K
*k*	29 W/mK	33 W/mK	8 W/mK
*ρ*	7850 kg/m^3^	7790 kg/m^3^	7120 kg/m^3^
*c*	450 J/kgK	476 J/kgK	443 J/kgK

It has been set that the hot runner is controlled at *T_p_* = 500 K while the *T_r_* is set as 320 K, considering the fairly high environmental temperature in injection molding facilities. In addition, the dimensions of the tested hot runner are specified in Table 2.

### 3.2. Stresses

**Figure 4:**
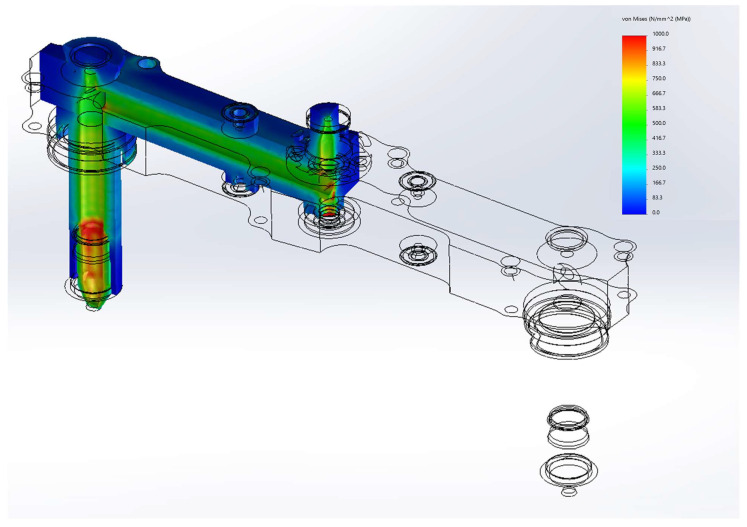
Overall stress level (von Mises stress) in the manifold and the nozzle.

Figure 4 shows the von Mises stress of the manifold assembly during the thermal and pressure loadings. The hot runner system is stressed since deformation is restrained by enclosing structures. In general, the outer plates are in a low-stress state, and the manifold has relatively even stress values but shows relatively high values.

A large value is shown near the nozzle, which is associated with a very large temperature gradient near the nozzle and the cross-sectional change of the runner. Although localized on the surface of the runner, the value is slightly above the known yield strength of AISI H13 steel at high temperatures (1200 MPa at 425 °C). It can also be seen that the locations in the supporting plate adjacent to the fastening points are all highly stressed. Inevitably stresses are concentrated on certain locations, which might render the hot runner further fatigued.

### 3.3. Deformations

**Figure 5:**
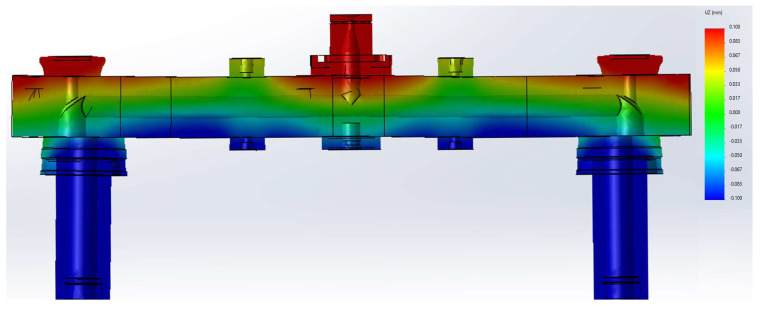
The deformation in the vertical direction (20 times magnified).

The hot runner system is stressed since deformation is restrained by enclosing structures. Figure 5 shows the deformation in the vertical direction alongside the nozzle alignments. As shown there, these restraints cause out-of-plane deformation. This is caused by the prevention of thermal deformation. The manifold desires to thermally expand uniformly in all directions. However, the bolting and the bushings in Figure 1 do not allow that. As can be seen in the figure, the displacement is quite high near the pin guide bush, insulation pad, and sprue bush. In the vicinity of those parts, the displacement in the z-direction is over 0.1 mm.

Inevitably, there is not enough structural support to prevent the bending. As shown in the figure, slight bending is also observed. The manifold near the nozzle is curved upward, forming a very mild cup-like shape. Note that the deformation in the figure is magnified 20 times.

**Figure 6:**
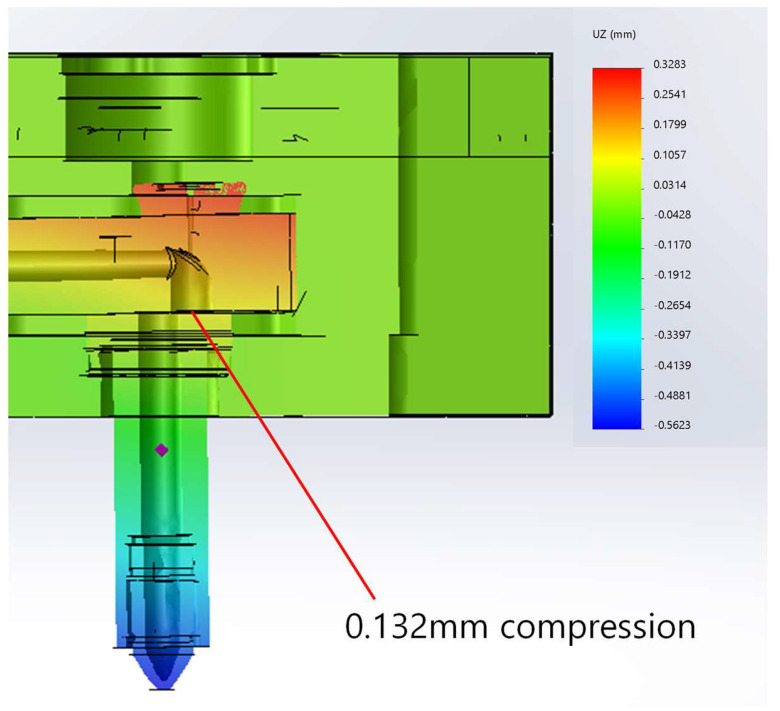
Magnified deflection of the hot runner system (100 times magnified).

To clearly visualize this, the deflection has been further magnified and shown in Figure 6. At the interface between the nozzle and the manifold, a compressive displacement of 0.132 mm has been observed. The nozzle and the manifold are misaligned, so the melt can possibly leak, and the gate pin cannot move freely if the situation aggravates. Here, two aspects should be considered. The first is the change in the thickness direction (*z*-direction in Figure 1), and the second is the change in the length (*y*-direction in Figure 1). The latter is very difficult to consider in real molds since the nozzle location adjustment is not viable. In other words, the initially assembled manifold has to be compressed or bent to allow the manifold to be straightened and stress-free under operational conditions. Thus, let us discuss the gap between the manifold and the nozzle bushing.

### 3.4. Gap

The design guide considering the thermal expansion of the pin guide bush or insulation pads is quite well described in the literature [2]. The important point is that at the point of the assembly, there should be an intentional gap that allows free thermal expansion of the manifold and bushings. Without the gap, huge stress can develop, and that stress can deform the assembly in an unwanted way. Note that specific molding techniques, such as micro-molding, necessitate a more accurate delivery of melt by minimizing deformations at the interfaces [24,25].

**Figure 7:**
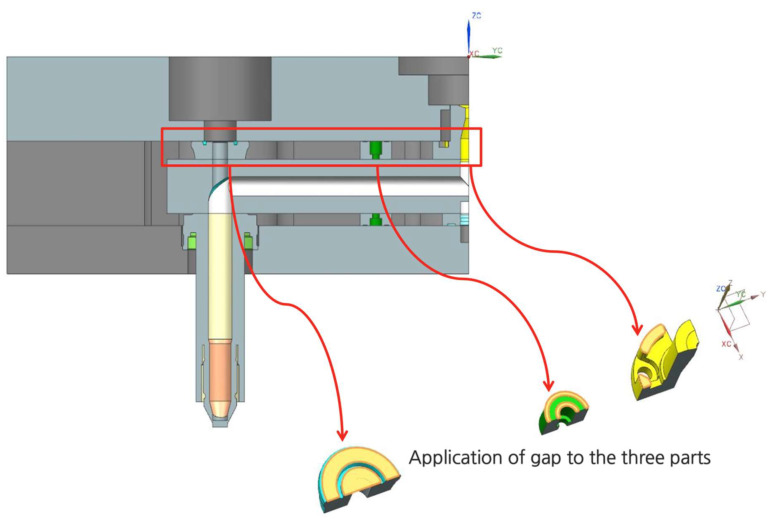
Application of gap by modifying the pin guide bush, insulation pad, and sprue bush.

**Figure 8:**
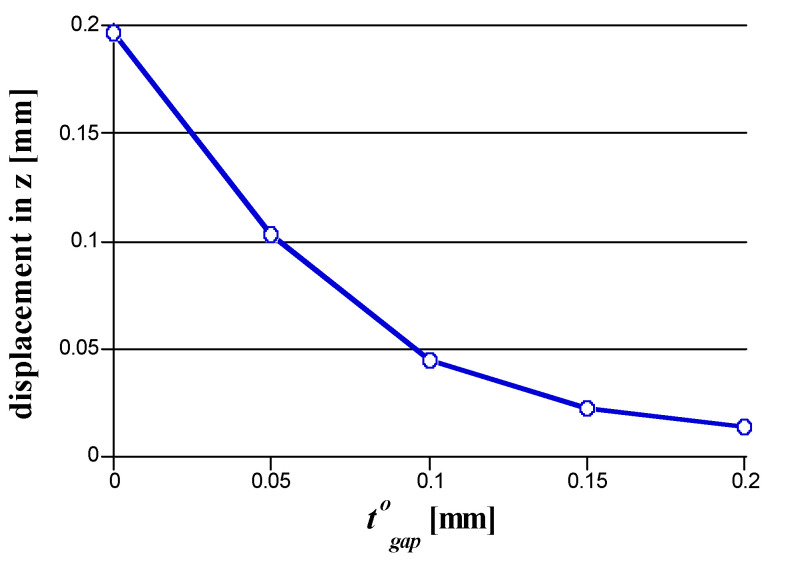
The displacement at the insulation pad center along with the gap.

Figure 7 shows how the gap is implemented in the hot runner assembly in this study. Figure 8 shows that when $t_{8}$ is reduced to $t_{8}-\delta$, the computed displacement components are reduced. Therefore, it is recommended that the gap be set to reduce such unwanted deformations. As shown in Figure 8, if the insulation pad thickness is reduced, the displacement would be significantly reduced. However, note that the displacement in *z* cannot be eliminated because it is affected by multi-dimensional effects such as horizontal thermal expansion.

The temperature rise of the space block can be regarded as $\Delta T_{2}\approx 0$. The manifold and nozzle bushing is subject to the temperature rise of $\Delta T_{0}=\Delta T_{5}=T_{p}-T_{r}$. That of the insulation pad can be evaluated as $\Delta T_{8}=\Delta T_{0}/2$. With the given $T_{p}$ and $T_{r}$, Equation (3) gives $t_{gap}^{o}=$ 0.133 mm. This result is quite close to the deformation shown in Figure 6. However, note that the numerical result considers the melt pressure while the result by Equation (3) does not. The deformation induced by melt pressure requires a comprehensive three-dimensional model.

Despite the necessity of the gap, according to mold builders, the manufacturers do not want to provide a hot runner with gaps. In theory, an intentional gap is required, but the design does not accommodate the gap in practice. The counterargument against the gap is that prestress can resist the possible force opening the connection between the nozzle and the manifold.

**Figure 9:**
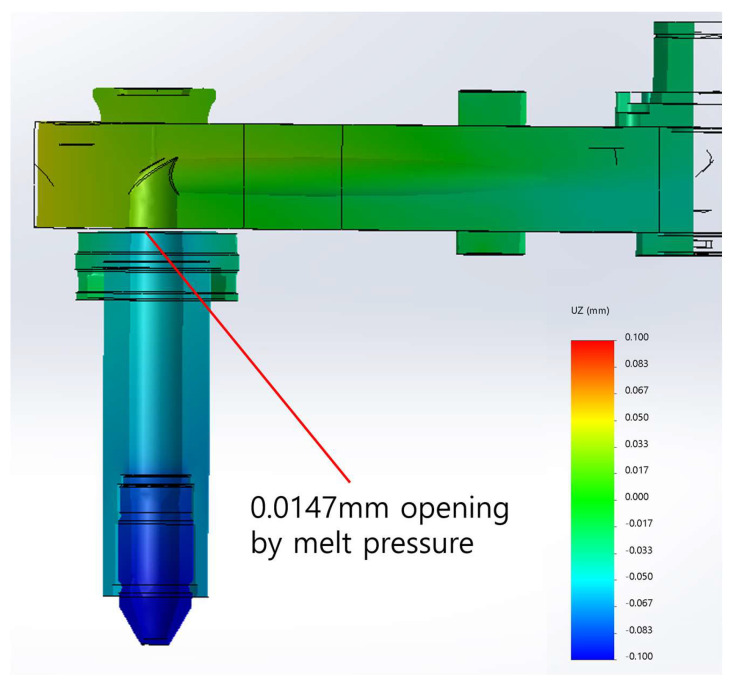
Gap estimated by pressure loading only (20 times magnified deformation for the view).

In a scenario where there is no thermal deformation and the deformation is solely caused by pressure, the resulting deformation depicted in Figure 9 has been achieved. The opening has been calculated as $t_{gap}^{p}=$0.0147 mm, which is an order of magnitude smaller than the thermal deformation in the opposite direction. As a result, this work suggests that the gap should be designed based on simulation results considering both the pressure and thermal loadings. Thus, this work suggests a design accommodating the gap by

(12) $$t_{gap}^{design}=t_{gap}^{o}-t_{gap}^{p}$$

**Figure 10:**
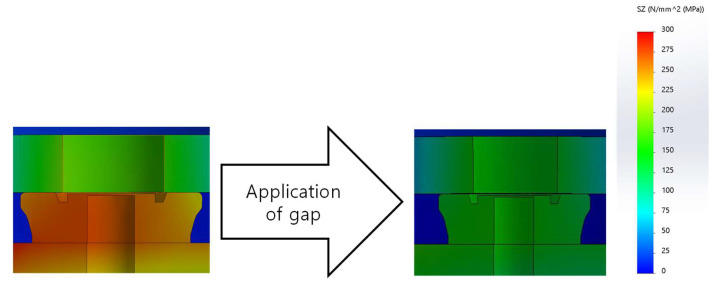
The change of the normal stress in z according to the application of the gap (from 0 mm to 0.132 mm).

When the gap by Equation (12) is applied, the stress around the pin guide bush is dramatically reduced, as shown in Figure 10.

### 3.5. Validation and Application

In this study, the theoretical numerical methodology needs experimental verification through the fabrication of actual hot runners. However, conducting experiments in high-temperature environments with assembled hot runners is challenging. A long-term usage method or an alternative approach involving the fabrication of experimental hot runners with exposed structures and observation of deformation and connection states could be considered, but this would incur significant costs. Despite the lack of experimental verification, the study’s results provide numerical evidence for previously theoretical design guidelines, such as Equation (1), enhancing confidence in their use.

The study assumes uniform maintenance of the surrounding thermal environment and the absence of heater failure as boundary conditions. Although the temperature distribution of the hot runner structure may not change significantly outside these conditions, actual hot runner failures often occur due to heater overheating, usually resulting from thermal bridges caused by resin leakage. Therefore, the design method proposed in this study has the potential to prevent control failures by addressing resin leakage. A simulation based on a more comprehensive model would offer detailed results that go beyond what this work provides. This is particularly relevant when considering a manifold that possesses intricate mechanical and thermal connections with the surrounding plates. When the deformation of surrounding plates becomes critical, such a model would be necessary to capture these complex interactions accurately.

## 4. Conclusions

This study focuses on simulating the deformation of a hot runner manifold and nozzle assembly. The deformation arises from thermal loading caused by temperature control, which is determined by calculating steady-state heat generation. Additionally, the melt pressure is accounted for, assuming a uniform pressure across the runner wall, gate, and sprue cross sections.

The simulation provides accurate predictions for the gap between the manifold and the nozzle bushing. While a simple calculation of thermal strain can serve as a reasonable approximation for design purposes, a simulation-based approach offers enhanced safety. It has been found that the deformation by the melt pressure does not exceed 12% of that by the thermal loading. This work has proposed how the gap can be set based on the simulation. By employing the gap, the stress can be reduced at the interface. As a result, the risk of catastrophic failures such as leaks or overheating can be mitigated. To facilitate the widespread adoption of the gap design, it is crucial to provide supporting experimental evidence.

## Figures and Tables

**Table 2:** Dimensions of the hot runner.

Parameter	Value (mm)	Parameter	Value (mm)	Parameter	Value (mm)
*t*_0_	45	*d*_0_	20	*s*_1_	15
*t*_1_	40	*d*_1_	22	*s*_2_	48
*t*_2_	70	*d*_2_	78	*s*_3_	15
*t*_3_	70	*d*_3_	14	*s*_4_	15
*t*_4_	16	*d*_4_	46	*s*_5_	66
*t*_5_	10	*d*_5_	30	*s*_6_	14
*t*_6_	14.3	*d*_6_	94	*s*_7_	32
*t*_7_	14.3	*d*_7_	30	*s*_8_	52
*t*_8_	15	*d*_8_	11	*s*_9_	6
*L*_0_	225	*d*_9_	6.5	*s*_10_	72
*L*_1_	85	*d*_10_	16.4	*s*_11_	125
